# Neonatal and young infant sepsis by Group B Streptococci and *Escherichia coli*: a single-center retrospective analysis in Germany—GBS screening implementation gaps and reduction in antibiotic resistance

**DOI:** 10.1007/s00431-020-03659-8

**Published:** 2020-05-23

**Authors:** Maren Doenhardt, Barbara Seipolt, Lars Mense, Jennifer Lucia Winkler, Alexander Thürmer, Mario Rüdiger, Reinhard Berner, Jakob Armann

**Affiliations:** 1 Department of Pediatrics, University Hospital and Medical Faculty Carl Gustav Carus, Technische Universität (TU) Dresden, Fetscherstraße 74, D-01307 Dresden, Germany; 2 Department of Gynecology and Obstetrics, University Hospital and Medical Faculty Carl Gustav Carus, Technische Universität (TU) Dresden, Fetscherstraße 74, D-01307 Dresden, Germany; 3 Institute of Medical Microbiology and Hygiene, Medical Faculty Carl Gustav Carus, Technische Universität (TU) Dresden, Fetscherstraße 74, D-01307 Dresden, Germany

**Keywords:** Neonatal Sepsis, *Group B Streptococcus*, *Escherichia coli*, Intrapartum antibiotic prophylaxis (IAP), Antibiotic resistance

## Abstract

The last nationwide surveillance study on neonatal and young infant sepsis due to Group B Streptococci (GBS) and *Escherichia coli* in Germany was conducted between 2009 and 2010. The aim of this study is to provide longitudinal epidemiological data on neonatal and young infant sepsis caused by GBS and *E. coli* to reevaluate existing data and to inform clinical decision-making. Every positive blood culture for GBS and *E. coli* within the first 90 days of life that occurred at our center from 2008 until 2018 was identified. The epidemiological, clinical, laboratory, and microbiological data of all affected patients were analyzed through retrospective chart review, along with the pathogen’s antimicrobial susceptibility results. In total, 106 episodes of neonatal sepsis were described; 31% (*n* = 33) being caused by GBS and 69% (*n* = 73) by *E. coli*; 87% of GBS early-onset disease (EOD) cases did not receive intrapartum antibiotic prophylaxis (IAP). Contrary to general trends, the proportion of resistant *E. coli* isolates decreased for all tested antibiotics over time. Coincidentally, antenatal antibiotic use beyond IAP during that period decreased significantly in our center.

*Conclusions*: (1) Data at our center suggests at least a regional implementation gap in GBS screening and IAP. (2) The decline in the resistance rate of *E. coli* for all antimicrobial substances might indicate that the reduction of prenatal antibiotics use is beneficial and that neonatal antibiotic stewardship programs should include pregnant women as well.

**Table:** 

**What is Known:** *• GBS screening and intrapartum antibiotic prophylaxis led to a 32%-reduction in GBS disease in Germany with a 0.75 (92:122) ratio of early-onset disease to late-onset disease in 2009–2010.* *• Prenatal antibiotic use might increase the risk of E. coli early-onset disease and antibiotic resistances.*	
**What is New:** *• The GBS early-onset disease rates were twice as high as those of late-onset disease, the ratio was 1.75 (21:12) in 2008–2018 at our institution. This suggests that there are at least regional implementation gaps in the antenatal GBS screening in Germany.* *• We found a decline in E. coli resistance rates over time for all antimicrobial substances. Reduction in use of prenatal antibiotics might be an explanation.*

**Electronic supplementary material:**

The online version of this article (10.1007/s00431-020-03659-8) contains supplementary material, which is available to authorized users.

## Introduction

Neonatal sepsis is a major cause of mortality and morbidity in infants [26]. *Escherichia coli* (*E. coli*) and Group B Streptococci (GBS, *Streptococcus agalactiae*) are the most common etiologic pathogens [16]. Antenatal GBS screening and intrapartum antibiotic prophylaxis (IAP) during labor have been shown to significantly decrease early-onset disease (EOD) caused by GBS [15, 23]. However, there are concerns that IAP might lead to an increase in neonatal sepsis caused by non-GBS pathogens, particularly *E. coli* [2, 19]. Germany has not yet adopted a universal GBS screening funded by the public health insurance, even though this screening has been recommended by periodically-reviewed national guidelines since 2000 [1]. Currently, no data is available on how widespread physicians are implementing this screening and IAP. However, the worldwide systematic review of IAP policies for the prevention of GBS disease by Le Doare et al. estimated a 60% coverage rate of IAP in Germany in 2017 [11].

It is important that physicians understand epidemiological changes in order to be able to perform informed clinical decision-making, especially regarding appropriate empirical antibiotic therapy. The German Neonatal Infection Surveillance Network (NeoKISS; “Krankenhaus-Infektions-Surveillance-System”) provides valuable data regarding longitudinal epidemiology; however, it only focuses on very low birth weight (VLBW), < 1500 g, premature infants [5]. A nationwide surveillance study on neonatal sepsis due to GBS and *E. coli* in Germany was last conducted between 2009 and 2010 [7, 23]. The aim of this retrospective data analysis is to update these results from 2010, describe the epidemiology, outcome, and bacterial resistance of neonatal infection due to GBS and *E. coli* over the past decade in a large German neonatal center.

## Material and methods

### Clinical data collection

Between January 2008 and December 2018, all patients with *E. coli* and GBS isolates from positive blood cultures within the first 90 days of life were identified at the Department of Pediatrics, University Hospital Dresden, TU Dresden, Germany. The detection of *E. coli* or GBS in blood cultures was the criterion for inclusion to the study. The epidemiological, clinical, and microbiological data of the infants and their mothers were collected through retrospective chart review.

Early-onset disease (EOD) was defined as infections occurring between the first and sixth day of life, infections occurring between the seventh and the 90th day of life were classified as late-onset disease (LOD).

Not every infant was born at our center, therefore not every infant’s and mother’s clinical history could be retrieved retrospectively. On this account some data is incomplete. Accordingly, not every of the 106 infant-mother-pairs offer complete data of antenatal microbiological swabs and use of IAP. IAP was considered adequate if there was documentation of at least 2 doses of penicillin G or ampicillin > 4 h before delivery.

However, for all infants weighing < 1500 g who were born at our institution from 2014 to 2018, data on antenatal antibiotic administration in women with pending premature birth at our center was available. This data of very low birth weight (VLBW) infants was collected separately within the perinatal conference at our center.

### Microbiological examination

Bacterial isolates from positive blood cultures (BACTEC, BD Diagnostics, Heidelberg, Germany) and screening swabs were identified based on colony morphology on Columbia blood agar using VITEK 2 (bioMérieux, Nürtingen, Germany), agglutination tests for ß-hemolytic streptococci (Pastorex Strep Kit, Bio-Rad, France), and, since 2011, MALDI-TOF MS (Bruker Daltonik, Bremen, Germany). Antimicrobial susceptibility testing was performed according to criteria of the European Committee on Antimicrobial Susceptibility Testing (EUCAST) of the European Society of Clinical Microbiology and Infectious Diseases (ESCMID), according the CLSI-standard (Clinical and Laboratory Standards Institute) [21]. Microbiological screening swabs were obtained per recommendation of the Commission for Hospital Hygiene and Infection Prevention at the Robert Koch- Institute in Germany (KRINKO). Ear swabs were taken from every newborn after birth. Throat and rectal swabs were obtained weekly for all patients.

### Laboratory

White blood cell count (WBC), immature to total neutrophil ratio (ITQ), C-reactive protein (CrP), and Interleukin-6 (IL-6) were analyzed as indications of bacterial infection. WBC 5 to 21 GPt/L, CrP < 10 mg/L, IL-6 < 150 pg/mL, and ITQ < 0.2 were considered typical values for healthy individuals.

### Statistical analyses

Results are expressed as the median value (range) for continuous variables and *n* (%) for categorical variables for the statistical analysis. The calculated percentage values always reflect the available data for that specific variable (Table 1).The binomial test was used to determine the male to female ratio and the effects of continuous covariates with the Kruskal-Wallis test. Fisher’s Exact test was used to determine categorical variables for the statistical analysis. *p* values ≤ 0.05 were ascertained to be statistically significant.

**Table 1:** Clinical characteristics of neonatal sepsis: Group B *Streptococcus* (GBS) vs. *Escherichia coli* (*E. coli*)

All (EOD + LOD) (*n* = 106)			GBS (*n* = 33)		*E. coli* (*n* = 73)		
			Values	NA	Values	NA	*p*
EOD(DOL1–6):LOD(DOL7–90)	ratio	(ratio)	21:12 (1.75)	0	38:35 (1.1)	0	NS
Male:female	ratio	(ratio)	20:13 (1.5)	0	41:32 (1.3)	0	NS
Age at time of onset (days)	median	(range)	2 (1–50)	0	6 (1–87)	0	NS
Gestational Age (weeks)	median	(range)	38 (26–41)	0	31 (23–41)	0	0.002
Preterm birth (<37 GA)	*n*	(%)	10 (30%)	0	55 (75%)	0	<0.001
Extreme preterm birth (<28 GA)	*n*	(%)	5 (15%)	0	23 (32%)	0	NS
Birthweight (g)	median	(range)	3210 (890–4370)	2	1430 (430–4660)	7	NS
Low Birthweight (<2500 g)	*n*	(%)	11 (33%)	0	55 (75%)	0	<0.001
Very low Birthweight (<1500 g)	*n*	(%)	5 (15%)	0	35 (48%)	0	0.001
Extremely low Birthweight (<1000 g)	*n*	(%)	3 (9%)	0	23 (32%)	0	0.01
C-section	*n*	(%)	11 (35%)	2	38 (58%)	8	NS
Multiple gestation	*n*	(%)	4 (12%)	0	10 (14%)	0	NS
Neonatal colonization at time of infection	*n*	(%)	18 (55%)	0	38 (52%)	0	NS
Mortality	*n*	(%)	2 (6%)	0	6 (8%)	0	NS
Meningitis	*n*	(%)	9 (27%)	0	6 (8%)	0	0.015
ICH	*n*	(%)	4 (12%)	0	27 (37%)	0	0.01
NEC	*n*	(%)	0 (0%)	0	6 (8%)	0	NS
BPD	*n*	(%)	2 (6%)	0	22 (30%)	0	0.006
Initial labs							
WBC <5 or > 21 GPt/L	*n*	(%)	14 (42%)	0	26 (36%)	0	NS
ITQ >0.2	*n*	(%)	23 (74%)	2	41 (64%)	9	NS
CrP >10 mg/L	*n*	(%)	16 (48%)	0	39 (55%)	2	NS
IL-6 > 1000 pg/mL	*n*	(%)	21 (81%)	7	30 (63%)	25	NS
All labs normal *	*n*	(%)	2 (8%)	7	8 (17%)	25	NS
Labs 36-72 h							
WBC <5 or > 21 GPt/L	*n*	(%)	9 (33%)	6	18 (33%)	19	NS
ITQ >0.2	*n*	(%)	4 (17%)	10	18 (37%)	24	NS
CrP >10 mg/L	*n*	(%)	19 (76%)	8	38 (78%)	24	NS
All labs normal **	*n*	(%)	2 (8%)	8	7 (14%)	24	NS
Maternal Age	median	(range)	30 (17–41)	1	20 (20–43)	2	NS
Only EOD (*n* = 59)			GBS (*n* = 21)		*E. coli* (*n* = 38)		
			Values	NA	Values	NA	*p*
Maternal WBC <4 or > 11 GPt/L at delivery	*n*	(%)	11 (92%)	9	20 (69%)	9	NS
Maternal CrP >10 mg/L at delivery	*n*	(%)	7 (88%)	13	12 (43%)	10	0.044
Amniotic swab same pathogen	*n*	(%)	7 (100%)	14	21 (81%)	12	NS
Vaginal colonization same pathogen	*n*	(%)	8 (80%)	11	12 (35%)	4	0.03
Rupture of membranes >18 h	*n*	(%)	5 (25%)	1	22 (59%)	1	0.03
IAP administration	*n*	(%)	2 (13%)	5	27 (77%)	3	<0.001
Antibiotic administration PROM	*n*	(%)	2 (13%)	5	27 (77%)	3	<0.001

Statistical analyses was formed with binomial test for male:female ratio, Kruskal-Wallis test for continuous covariates, and Fisher’s exact tests for categorical variables. *p* values of ≤ 0.05 were deemed to be significant.

*EOD* Early-onset disease (day of life 1–6d), *LOD* Late-onset disease (day of life 7–90d), *GA* Gestational age, *NA* Number of cases for which data were not available, *NS* Not significant (*p* > 0.05), *ICH* Intracerebral hemorrhage, *NEC* Necrotizing enterocolitis, *BPD* Bronchopulmonary dysplasia, *WBC* White blood cells, *ITQ* immature/total quotient, *CrP* C-reactive protein, *IAP* Intrapartum antibiotic prophylaxis, *PROM* Premature rupture of membranes.

* WBC 5–21, ITQ < 0.2, CrP <10, IL-6 < 150

** WBC 5–21, ITQ < 0.2, CrP <10

## Results

### Epidemiology

**Fig. 1:**
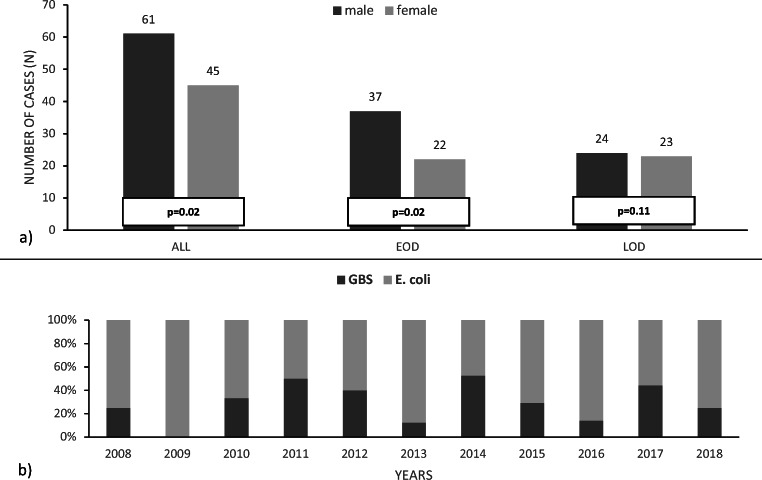
Ratios. **a** Male: female ratio in all cases (*p* = 0.02), early-onset disease (*p* = 0.02), and late-onset disease (*p* = 0.11). **b** GBS: *E. coli* ratio over 10-year study period (2008–2018)

A total of 106 episodes of blood culture proven neonatal and young infant sepsis were identified during the study period, with 31% (*n* = 33) caused by GBS and 69% (*n* = 73) by *E. coli*. The infection rate was higher for male (*n* = 61, 58%) than female infants (*n* = 45, 42%; *p* = 0.02). There was no significant difference found between the male/female ratio for GBS (m:f = 20:13; 1.5) and *E. coli* (41:32; 1.3). However, the predominance of male infants could be detected only for EOD (37:22; 1.8; *p* = 0.02) and not for LOD (24:23; 1.05; *p* = 0.1) (Fig. 1a).

**Fig. 2:**
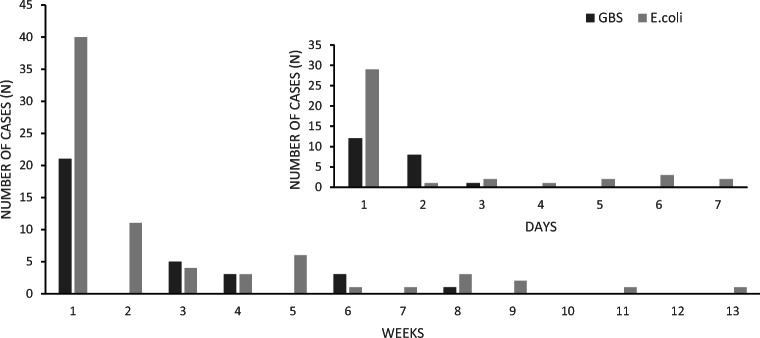
Age at time of diagnosis (day/week of life)

Annually, the ratio between *E. coli* and GBS neonatal sepsis ranged from 1:1 to 7:1, without any identifiable trend (Fig. 1b). Figure 2 shows the infant’s age at the time of diagnosis for each case. Two thirds of GBS cases were EOD whereas *E. coli* was evenly distributed throughout EOD and LOD.

GBS blood stream infections were more common in full-term infants (*n* = 23, 70%) than *E. coli* (*n* = 18, 25%; *p* = <0.001); even when analyzed for EOD and LOD separately, this did not change (EOD p = <0.001 / LOD *p* = 0.02) (A1 Tables 1, 2, and 3).

The overall number of C-sections did not differ between GBS and *E. coli*. Focusing only on LOD, infants with *E. coli* infection (*n* = 16, 59%), additional to the fact that they were more preterm (*p* = 0.02), were more often born via C-section than infants with GBS infection (*n* = 1, 10%; *p* = 0.01) (A1 Table 3).

During routine microbiological screening swabs as recommended by the KRINKO, neonatal colonization with the pathogen causing invasive disease did not differ between GBS (55%) and *E. coli* (52%) at the time of infection onset. Approximately one-third of the infants were colonized with another pathogen than the one causing invasive disease (A1 Tables 1, 2, and 3).

### Outcome

Regarding all EOD and LOD cases, there was no detectable difference between GBS (*n* = 2, 6%) and *E. coli* (*n* = 6, 8%) mortality. Significantly more GBS infected infants suffered from meningitis (GBS *n* = 9, 27%; *E. coli n* = 6, 8%; *p* = 0.015). Specifically, LOD with GBS occurred with 58% (*n* = 7) of meningitis cases, in contrast to 9% (*n* = 3) for *E. coli* (*p* = 0.001) (A1 Table 3). Overall, complications like intracerebral hemorrhage (ICH) and bronchopulmonary dysplasia (BPD) affected more *E. coli*- than GBS-infected children (*p*(ICH) = 0.01; *p*(BPD) = 0.006) (Table 1).

### Biomarkers

Laboratory studies at disease onset and after 36–72 h were analyzed. The data for WBC, ITQ, CrP, and IL-6 is summarized in Table 1 (A1 Tables 1, 2, 3, and 4). At the time of infection, IL-6 and ITQ were generally the most sensitive markers; however, in subsequential labs, CrP was the most sensitive marker. Initially, 8% of infants with GBS disease and 17% of infants with *E. coli* disease showed no elevated blood markers indicating a bacterial infection. On repeated evaluation, 8% of GBS and 14% of *E. coli* infections continued to lack abnormalities in laboratory studies despite proven bacteremia. Only 17% (10 of 59) of all EOD (2 of 21 GBS EOD, 8 of 38 *E. coli* EOD) had IL-6 levels < 150 pg/mL; 90% (9/10) of these infants were preterm. Only in GBS LOD, all patients presented at least one abnormal blood marker.

There were no statistically significant differences between *E. coli* and GBS infection regarding any of the analyzed biomarkers. Between first and second blood samples, however, the increase in CrP as well as the decrease in ITQ were significant (*p*(CrP) = 0.0005 and *p*(ITQ) = 0.0001) (A2 Table 5).

### Maternal factors

The results of maternal laboratory tests were analyzed for EOD cases. The proportion of increased CrP at delivery (> 10 mg/L) was significantly higher in women giving birth to children with GBS (*n* = 7, 80%) compared with children with *E. coli* sepsis (*n* = 12, 43%; *p* = 0.044).

Prepartal vaginal swabs and amniotic membrane swabs taken at time of the C-section were compared with the infants’ blood culture results. Results of prepartal vaginal or amniotic swabs were not available for all women. Amniotic swabs and vaginal swabs were available for nearly every C-section that was performed at our center. Regarding all women, prepartal vaginal swabs, that were taken a few days or hours before delivery, were available in 67 of 106 cases. Focusing only on EOD, prepartal vaginal swabs were available in 10 of 21 GBS and 34 of 38 *E. coli* cases (Table 1). In EOD cases, 80% of mothers (whose children subsequently developed GBS disease) were vaginally colonized with GBS before birth whereas in cases of *E. coli*, only 35% were vaginally colonized (*p* = 0.03). In C-sectioned women, amniotic swabs demonstrated detection rates of 100% for GBS and 81% for *E. coli* with regard to diseased infants.

Only two infants (13% of GBS EOD cases), both preterm, developed early onset GBS disease despite intrapartum prophylaxis. The rate of premature rupture of membranes (PROM) > 18 h was significantly higher in EOD *E. coli* (*n* = 21, 59%) than EOD GBS cases (*n* = 5, 25%) (*p* = 0.03). Antibiotic administration for PROM was significantly more frequent in mothers whose children developed *E. coli* EOD (*n* = 20, 57%) compared with GBS (*n* = 2; 13%; *p* = 0.001) (Table 1; A1 Table 2).

### Antimicrobial resistance rates

**Fig. 3:**
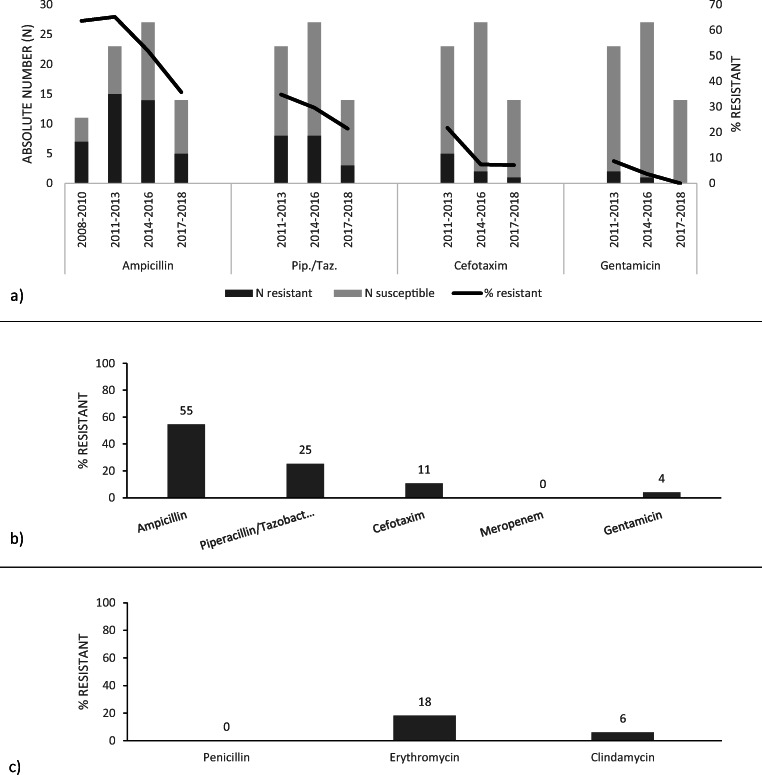
Antimicrobial resistance rate of **a** *E. coli* over 10-year study period (2008–2018), **b** *E. coli* overall, and **c** GBS overall

All GBS isolates were susceptible to penicillin, 6% were resistant to clindamycin, and 18% were resistant to erythromycin (Fig. 3c).

The proportion of resistant *E. coli* isolates decreased during the 10-year period for all tested antibiotics. Ampicillin resistance decreased from 64 to 36% (*p* = 0.11), piperacillin-tazobactam resistance from 35 to 21% (*p* = 0.47), cefotaxime resistance from 21 to 7% (*p* = 0.37), and gentamicin resistance from 9 to 0% (*p* = 0.51). Overall 55% of *E. coli* isolates were resistant to ampicillin, 25% to piperacillin-tazobactam, 11% to cefotaxime, and 4% to gentamicin. All isolates were susceptible to meropenem (Fig. 3a, b).

**Fig. 4:**
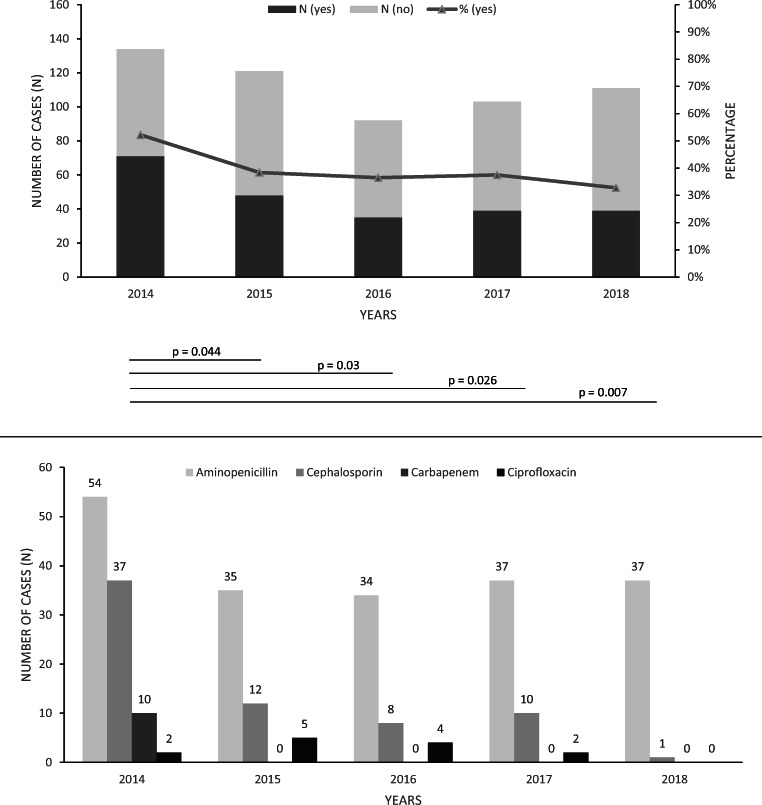
Antenatal antibiotic administration in women with a pending premature birth, regarding all premature infants (weighing < 1500 g) born at our institution from 2014 to 2018

From 2014 to 2018, overall antenatal antibiotic use in women with a pending premature birth decreased (52 vs. 33%, *p* = 0.007), with significantly reduced rates of carbapenems and cephalosporins (Fig. 4).

## Discussion

At our center, GBS caused sepsis predominately in full-term neonates whereas *E. coli* caused disease mainly in preterm infants. These results are consistent with the existing literature [16]. The 1:2 infection rate of GBS to *E. coli* is contrary to the 2:1 infection rate found in a nationwide surveillance study in Germany in 2009–2010 [10]. This can be explained by an overrepresentation of premature infants in the selection sample of our tertiary perinatal center.

The observed mortality from GBS disease in 6% of patients in our study is consistent with previous reports [6]. The *E. coli* mortality rate in our study was significantly lower than that found in previous studies [13, 16]. Nevertheless, 75% of infants with *E. coli* sepsis were preterm, and circa 50% were below 1500 g at birth and, therefore, critically vulnerable.

Consistent with current literature, 60% of patients with GBS LOD in our study had meningitis, however, also 8–10% of infants with GBS EOD or *E. coli* suffered from meningitis [8]. Given the therapeutic and prognostic consequences, this warrants evaluation of all bacteremic infants for meningitis regardless of onset of disease.

The higher incidence of ICH and BPD in *E. coli* sepsis most probably is due to the higher proportion of premature infants with *E. coli* sepsis. This difference is no longer statistical significant after stratifying for gestational age (A1 Table 4).

We confirmed the reasonable high sensitivity of ITQ and IL-6 at the time of diagnosis, indicating that biomarkers indeed may help to rule out neonatal and young infant sepsis and thereby help to reduce the well-described antibiotic overuse in this age group [17].

However, there was no significant, detectable increase in CrP levels before 36–72 h after disease-onset. [18]. Interestingly, even after subsequent evaluation, 8–14% of infants with proven bacteremia did not have any abnormal biomarkers, which emphasizes the importance of blood culture diagnostics in suspected bloodstream infection.

Maternal biomarkers seem to be pathogen-dependent, with higher positive rates in GBS than *E. coli* EOD. This suggests that negative laboratory studies should never be seen to definitely rule out sepsis, in particular not in preterm infants with a high risk of *E. coli* sepsis.

Universal antenatal GBS screening and IAP) in the United States led to a six-fold decrease in the incidence of EOD, and currently, approximately equal rates of EOD and LOD [15]. Wicker et al. described the development of GBS disease after publication of the guidelines for culture-based screening for GBS colonization in Germany. A 32% reduction in GBS disease incidence was detected. The ratio of early-onset disease to late-onset disease reversed from 1.52 (206:136) in 2001–2003 to 0.75 (92:122) in 2009–2010 [23]. In contrast, our study shows a ratio of 1.75 (21:12) with GBS EOD rates that are twice as high as those for LOD. This suggests that there are at least regional implementation gaps in the antenatal GBS screening in Germany. A universal screening program would therefore lead to a further reduction in infant morbidity and mortality from GBS. The fact that only two infants with GBS EOD received the IAP further substantiates this point. The most likely explanation for the IAP failure in those children is the fact that they were born very premature at 27 weeks gestational age making them more susceptible for infections.

The high burden of *E. coli* disease in premature infants highlights the need for effective prevention. Administration of broad-spectrum antibiotics based on maternal microbiological sampling has been suggested [12, 24, 25]. However, in contrast to GBS, *E. coli* can only be detected in one-third of the respective EOD cases in prepartal vaginal swabs, making it difficult to identify patients at risk for *E. coli* sepsis. More importantly, there is data that suggest that antenatal antibiotic use might increase the risk of *E. coli* EOD [22]. The high prevalence of *E. coli* in EOD infants that were prenatally exposed to antibiotics in our study substantiates these findings and calls this approach into question.

Antimicrobial resistance rates for GBS were overall low and consistent with results from a previous nationwide surveillance study in Germany in 2009–2010 [3, 7, 23]. Resistance rates for *E. coli*, on the other hand, were cumulatively higher than those reported in this previous study, especially for ampicillin and third-generation cephalosporins. In addition, we found a decline in resistance rates over time for all antimicrobial substances in our study, and notably for ampicillin. An explanation might be the reduction in use of prenatal antibiotics in our obstetrics and gynecology department, especially broad-spectrum antibiotics such as cephalosporins and carbapenems. This coincides with the decrease in resistant *E. coli* in neonatal sepsis, contrary to the previously published findings; Dona et al. as well reported about a decrease in extended spectrum beta-lactamase (ESBL) producers *E. coli* [3, 4, 9, 14, 20]. Nevertheless, these results are due to the implementation of pediatric antibiotic stewardship programs (ASP). Our data points to the importance of including pregnant women in neonatal ASPs as well.

Our study is limited by its retrospective design leading to incomplete data especially in mothers who were not treated at our institution. In addition, we provide regional data only from a single center that might not necessarily reflect the situation in other parts of Germany accurately. We feel confident though that our findings warrant additional studies in Germany in regard to the nationwide implementation of screening and prophylaxis and the development of resistance rates.

## Conclusion

Given the documented regional implementation gaps of the risk-based GBS screening further studies are needed to evaluate the nationwide implementation of current guidelines. A universal GBS screening could reduce infant mortality and morbidity in Germany.

Antenatal long-term administration of broad-spectrum antibiotics to women with pending premature birth might increase the incidence of *E. coli* EOD and leads to antimicrobial resistance. Rational antenatal antibiotic prescription might help reduce the problem of multi-drug resistance in neonatal and young infant sepsis. It is important that neonatal ASPs include pregnant women as well.

## Electronic supplementary material

ESM 1 (PDF 269 kb)

ESM 2 (PDF 123 kb)

## Abbreviations

ASP: Antibiotic stewardship program
BPB: Bronchopulmonary dysplasia
CrP: C-reactive protein
*E. coli*: *Escherichia coli*
EOD: Early-onset disease
GBS: *Group B Streptococcus*
IAP: Intrapartum antibiotic prophylaxis
ICH: Intracerebral hemorrhage
IL-6: Interleukin-6
ITQ: Immature to total neutrophil ratio (quotient)
LOD: Late-onset disease
N: Number
PROM: Premature rupture of membranes
VLBW: Very low birth weight
WBC: White blood cell count
