# Supplementary material for: Neonatal and young infant sepsis by Group B Streptococci and Escherichia coli: a single-center retrospective analysis in Germany—GBS screening implementation gaps and reduction in antibiotic resistance
Source: Eur J Pediatr. 2020 May 23;179(11):1769–77. doi: 10.1007/s00431-020-03659-8 (PMC7547982; doi:10.1007/s00431-020-03659-8)
Supplement: Supplementary file 1 — (PDF 269 kb) [file 431_2020_3659_MOESM1_ESM.pdf]

TABLE I. Clinical characteristics of neonatal sepsis (early- and late-onset disease): group B streptococcus (GBS) vs. Escherichia coli (E. coli)

			GBS (N = 33)		E. coli (N = 73)		
			Values	NA	Values	NA	p
EOD(DOL1-6):LOD(DOL7-90)	ratio	(ratio)	21:12 (1.75)	0	38:35 (1.1)	0	NS
Male:female	ratio	(ratio)	20:13 (1.5)	0	41:32 (1.3)	0	NS
Age of time of onset (days)	median	(range)	2 (1-50)	0	6 (1-87)	0	NS
Gestational Age (weeks)	median	(range)	38 (26-41)	0	31 (23-41)	0	0.002
Preterm birth (<37 GA)	n	(%)	10 (30%)	0	55 (75%)	0	<0.001
Extreme preterm birth (<28 GA)	n	(%)	5 (15%)	0	23 (32%)	0	NS
Birthweight (g)	median	(range)	3210 (890-4370)	2	1430 (430-4660)	7	NS
Low Birthweight (<2500g)	n	(%)	11 (33%)	0	55 (75%)	0	<0.001
Very low Birthweight (<1500g)	n	(%)	5 (15%)	0	35 (48%)	0	0.001
Extremely low Birthweight (<1000g)	n	(%)	3 (9%)	0	23 (32%)	0	0.01
C-section	n	(%)	11 (35%)	2	38 (58%)	8	NS
Multiple gestation	n	(%)	4 (12%)	0	10 (14%)	0	NS
Neonatal colonization at time of infection	n	(%)	18 (55%)	0	38 (52%)	0	NS
Mortality	n	(%)	2 (6%)	0	6 (8%)	0	NS
Meningitis	n	(%)	9 (27%)	0	6 (8%)	0	0.015
ICH	n	(%)	4 (12%)	0	27 (37%)	0	0.01
NEC	n	(%)	0 (0%)	0	6 (8%)	0	NS
BPD	n	(%)	2 (6%)	0	22 (30%)	0	0.006
**Initial labs**							
WBC (GPt/L) median	median	(range)	12.2 (2-33)	0	9.12 (0-39)	0	NS
WBC <5 or >21 GPt/L	n	(%)	14 (42%)	0	26 (36%)	0	NS
ITQ >0.2	n	(%)	23 (74%)	2	41 (64%)	9	NS
CrP (mg/L)	median	(range)	9.9 (0-150)	0	13.5 (0-294)	2	NS
CrP >10mg/L	n	(%)	16 (48%)	0	39 (55%)	2	NS
IL-6 (pg/mL)	median	(range)	5000 (26-162550)	7	2061.5 (4-112499)	25	NS
IL-6 >1000pg/mL	n	(%)	21 (81%)	7	30 (63%)	25	NS
All labs normal *	n	(%)	2 (8%)	7	8 (17%)	25	NS
**Labs 36-72h**							
WBC (GPt/L) median	median	(range)	14.11 (2-57)	6	13.89 (0-52)	19	NS
WBC <5 or >21 GPt/L	n	(%)	9 (33%)	6	18 (33%)	19	NS
ITQ >0.2	n	(%)	4 (17%)	10	18 (37%)	24	NS
CrP (mg/L)	median	(range)	37.9 (2-315)	8	52.3 (0-292)	24	NS
CrP >10mg/L	n	(%)	19 (76%)	8	38 (78%)	24	NS
All labs normal **	n	(%)	2 (8%)	8	7 (14%)	24	NS
Catecholamines	n	(%)	10 (30%)	0	23 (32%)	0	NS
FFP transfusion	n	(%)	9 (27%)	0	18 (25%)	0	NS
RBC transfusion	n	(%)	9 (27%)	0	32 (44%)	0	NS
Maternal Age	median	(range)	30 (17-41)	1	20 (20-43)	2	NS

TABLE II. Clinical characteristics of early-onset disease (EOD): group B streptococcus (GBS) vs. Escherichia coli (E. coli)

			GBS (N = 21)		E. coli (N = 38)		
			Values	NA	Values	NA	p
Male:female	ratio	(ratio)	15:6 (2.5)	0	22:16 (1.4)	0	NS
Age of time of onset (days)	median	(range)	1 (1-3)	0	1 (1-6)	0	NS
Gestational Age (weeks)	median	(range)	37 (26-41)	0	31 (23-41)	0	0.006
Preterm birth (<37 GA)	n	(%)	8 (38%)	0	34 (89%)	0	<0.001
Extreme preterm birth (<28 GA)	n	(%)	5 (24%)	0	11 (29%)	0	NS
Birthweight (g)	median	(range)	3250 (890-4320)	0	1570 (560-4660)	0	NS
Low Birthweight (<2500g)	n	(%)	7 (33%)	0	35 (92%)	0	<0.001
Very low Birthweight (<1500g)	n	(%)	5 (24%)	0	18 (47%)	0	NS
Extremely low Birthweight (<1000g)	n	(%)	3 (14%)	0	10 (26%)	0	NS
C-section	n	(%)	10 (48%)	0	22 (58%)	0	NS
Multiple gestation	n	(%)	2 (10%)	0	3 (8%)	0	NS
Neonatal colonization at time of infection	n	(%)	16 (76%)	0	26 (68%)	0	NS
Mortality	n	(%)	1 (5%)	0	4 (11%)	0	NS
Meningitis	n	(%)	2 (10%)	0	3 (8%)	0	NS
ICH	n	(%)	4 (19%)	0	17 (45%)	0	NS
NEC	n	(%)	0 (0%)	0	2 (5%)	0	NS
BPD	n	(%)	2 (10%)	0	10 (26%)	0	NS
**Initial labs**							
WBC (GPt/L) median	median	(range)	12.65 (3-33)	0	9.11 (1-39)	0	NS
WBC <5 or >21 GPt/L	n	(%)	8 (38%)	0	13 (34%)	0	NS
ITQ >0.2	n	(%)	13 (65%)	1	20 (61%)	5	NS
CrP (mg/L)	median	(range)	5.4 (0-105)	0	2.3 (0-)	1	NS
CrP >10mg/L	n	(%)	6 (29%)	0	11 (30%)	1	NS
IL-6 (pg/mL)	median	(range)	5000 (26-162550)	2	2384 (4-)	2	NS
IL-6 >1000pg/ml	n	(%)	16 (84%)	2	23 (64%)	2	NS
All labs normal *	n	(%)	2 (11%)	2	5 (16%)	7	NS
**Labs 36-72h**							
WBC (GPt/L) median	median	(range)	21.71 (6-57)	5	13.07 (4-44)	12	NS
WBC <5 or >21 GPt/L	n	(%)	8 (50%)	5	10 (38%)	12	NS
ITQ >0.2	n	(%)	4 (27%)	6	7 (28%)	13	NS
CrP (mg/L)	median	(range)	20.9 (2-109)	6	18.1 (1-292)	13	NS
CrP >10mg/L	n	(%)	9 (60%)	6	17 (68%)	13	NS
All labs normal **	n	(%)	2 (13%)	6	5 (21%)	14	NS
Maternal WBC (GPt/L) at delivery	median	(range)	20.12 (10-23)	9	13.47 (7-25)	9	NS
Maternal WBC <4 or >11 GPt/L at delivery	n	(%)	11 (92%)	9	20 (69%)	9	NS
Maternal CrP (mg/L) at delivery	median	(range)	23.3 (2-98)	13	6.7 (1-180)	10	NS
Maternal CrP >10mg/L at delivery	n	(%)	7 (88%)	13	12 (43%)	10	0.044
Maternal Age	median	(range)	30 (17-41)	0	30 (20-43)	0	NS
Amniotic swab same pathogen	n	(%)	7 (100%)	14	21 (81%)	12	NS
Vaginal colonization same pathogen	n	(%)	8 (80%)	11	12 (35%)	4	0.03
Rupture of membranes >18h	n	(%)	5 (25%)	1	22 (59%)	1	0.03
IAP administration	n	(%)	2 (13%)	5	27 (77%)	3	<0.001
Antibiotic administration PROM	n	(%)	2 (13%)	5	20 (57%)	3	<0.001

TABLE III. Clinical characteristics of late-onset disease (LOD): group B streptococcus (GBS) vs. Escherichia coli (E. coli)

			GBS (N = 12)		E. coli (N = 35)		
			Values	NA	Values	NA	p
Male:female	ratio	(ratio)	5:7 (0.7)	0	19:16 (1.2)	0	NS
Age of time of onset (days)	median	(range)	24 (17-50)	0	22 (7-87)	0	NS
Gestational Age (weeks)	median	(range)	38.5 (34-40)	0	32 (23-40)	0	0.02
Preterm birth (<37 GA)	n	(%)	2 (17%)	0	21 (60%)	0	0.02
Extreme preterm birth (<28 GA)	n	(%)	0 (0%)	0	12 (34%)	0	0.02
Birthweight (g)	median	(range)	2930 (2010-4730)	2	1020 (430-4120)	7	NS
Low Birthweight (<2500g)	n	(%)	4 (40%)	2	20 (71%)	7	NS
Very low Birthweight (<1500g)	n	(%)	0 (0%)	2	17 (61%)	7	<0.001
Extremely low Birthweight (<1000g)	n	(%)	0 (0%)	2	13 (46%)	7	0.008
C-section	n	(%)	1 (10%)	2	16 (59%)	8	0.01
Multiple gestation	n	(%)	2 (22%)	3	7 (23%)	5	NS
Neonatal colonization at time of infection	n	(%)	2 (17%)	0	25 (71%)	0	0.002
Mortality	n	(%)	1 (8%)	0	2 (6%)	0	NS
Meningitis	n	(%)	7 (58%)	0	3 (9%)	0	0.001
ICH	n	(%)	0 (0%)	0	10 (29%)	0	NS
NEC	n	(%)	0 (0%)	0	4 (11%)	0	NS
BPD	n	(%)	0 (0%)	0	12 (34%)	0	0.02
**Initial labs**							
WBC (GPt/L) median	median	(range)	8.19 (2-25)	0	9.12 (0-33)	0	NS
WBC <5 or >21 GPt/L	n	(%)	6 (50%)	0	13 (37%)	0	NS
ITQ >0.2	n	(%)	10 (91%)	1	21 (68%)	4	NS
CrP (mg/L)	median	(range)	69.1 (1-150)	0	64.5 (1-170)	1	NS
CrP >10mg/L	n	(%)	10 (83%)	0	27 (79%)	1	NS
IL-6 (pg/ml)	median	(range)	5462.6 (318-98656)	5	1774 (25-55580)	23	NS
IL-6 >1000pg/ml	n	(%)	5 (71%)	5	7 (58%)	23	NS
All labs normal *	n	(%)	0 (0%)	5	1 (8%)	23	NS
**Labs 36-72h**							
WBC (GPt/L) median (range)	median	(range)	13.59 (2-21)	1	13.9 (0-52)	7	NS
WBC <5 or >21 GPt/L	n	(%)	1 (9%)	1	8 (29%)	7	NS
ITQ >0.2	n	(%)	0 (0%)	4	11 (46%)	11	0.03
CrP (mg/L)	median	(range)	69.65 (11-315)	2	92.1 (0-287)	11	NS
CrP >10mg/L	n	(%)	10 (100%)	2	21 (88%)	11	NS
All labs normal **	n	(%)	0 (0%)	4	2 (10%)	15	NS
Maternal Age	median	(range)	28 (19-38)	1	20 (30-43)	2	NS

TABLE IV. Clinical characteristics of neonatal sepsis (early- and late onset disease): group B streptococcus (GBS) vs. Escherichia coli (E. coli) < 37 weeks of gestational age (GA)

			GBS (N = 10)		E. coli (N = 73)		
			Values	NA	Values	NA	p
Male:female	ratio	(ratio)	8:2 (4.0)	0	26:29 (0.9)	0	NS
Age of time of onset (days)	median	(range)	1 (1-21)	0	2 (1-87)	0	NS
Gestational Age (weeks)	median	(range)	28 (26-35)	0	28 (23-34)	0	NS
Preterm birth (<37 GA)	n	(%)	10 (100%)	0	55 (100%)	0	NS
Extremly preterm birth (<28 GA)	n	(%)	5 (50%)	0	23 (42%)	0	NS
Birthweight (g)	median	(range)	1370 (990-2940)	0	1100 (430-4000)	7	NS
Low Birthweight (<2500g)	n	(%)	9 (90%)	0	54 (98%)	0	NS
Very low Birthweight (<1500g)	n	(%)	5 (50%)	0	34 (62%)	0	NS
Extremely low Birthweight (<1000g)	n	(%)	2 (20%)	0	23 (42%)	0	NS
C-section	n	(%)	6 (60%)	0	36 (65%)	8	NS
Multiple gestation	n	(%)	1 (10%)	0	9 (16%)	0	NS
Neonatal colonization at time of infection	n	(%)	6 (60%)	0	37 (67%)	0	NS
Mortality	n	(%)	1 (10%)	0	5 (9%)	0	NS
Meningitis	n	(%)	2 (20%)	0	3 (5%)	0	NS
ICH	n	(%)	4 (40%)	0	25 (46%)	1	NS
NEC	n	(%)	0 (0%)	0	6 (11%)	1	NS
BPD	n	(%)	2 (20%)	0	22 (41%)	1	NS
**Initial labs**							
WBC (GPt/L) median	median	(range)	4 (2-23)	0	8.83 (0-39)	0	NS
WBC <5 or >21 GPt/L	n	(%)	6 (40%)	0	20 (36%)	0	NS
ITQ >0.2	n	(%)	5.5 (67%)	1	33 (67%)	6	NS
CrP (mg/L)	median	(range)	4 (0-114)	0	6.45 (0-202)	1	NS
CrP >10mg/L	n	(%)	5000 (40%)	0	24 (44%)	1	NS
IL-6 (pg/mL)	median	(range)	8 (26-50000)	0	1169 (4-112499)	12	NS
IL-6 >1000pg/mL	n	(%)	1 (89%)	0	27 (63%)	12	NS
All labs normal *	n	(%)	1 (10%)	1	5 (10%)	3	NS
**Labs 36-72h**							
WBC (GPt/L) median	median	(range)	4 (6-57)	2	14.07 (0-52)	14	NS
WBC <5 or >21 GPt/L	n	(%)	1 (50%)	2	16 (39%)	14	NS
ITQ >0.2	n	(%)	9.1 (13%)	2	15 (38%)	16	NS
CrP (mg/L)	median	(range)	2 (2-248)	4	23.8 (0-292)	18	NS
CrP >10mg/L	n	(%)	1 (33%)	4	26 (70%)	18	NS
All labs normal **	n	(%)	1 (14%)	3	7 (17%)	14	NS
Maternal Age	median	(range)	33 (26-41)	0	30 (20-43)	0	NS
Amniotic swab same pathogen	n	(%)	5 (100%)	5	20 (50%)	15	NS
Vaginal colonization same pathogen	n	(%)	5 (71%)	3	15 (31%)	7	NS
Rupture of membranes >18h	n	(%)	6 (60%)	0	27 (50%)	1	NS

EOD	Early-onset disease (day of life 1-6d)	NEC	Necrotizing enterocolitis	FFP	Fresh Frozen Plasma
LOD	Late-onset disease (day of life 7-90d)	BPD	Bronchopulmonary dysplasia	RBC	Red blood cells
GA	Gestational age	WBC	White blood cells	IAP	Intrapartum antibiotic prophylaxis
NA	Number of cases for which data were not available	ITQ	immature/total quotient	PROM	Premature rupture of membranes
NS	Not significant (p>0.05)	CrP	C-reactive protein	*	WBC 5-21, ITQ<0.2, CrP <10, IL-6 <150
ICH	Intracerebral hemorrhage	IL-6	Interleukin-6	**	WBC 5-21, ITQ<0.2, CrP <10

Statistical analyses was formed with binomial test for male:female ratio, Kruskal-Wallis test for continuous covariates and Fisher's exact tests for categorical variables.
P-values of ≤0.05 were deemed to be significant.
