# Supplementary material for: Neonatal and young infant sepsis by Group B Streptococci and Escherichia coli: a single-center retrospective analysis in Germany—GBS screening implementation gaps and reduction in antibiotic resistance
Source: Eur J Pediatr. 2020 May 23;179(11):1769–77. doi: 10.1007/s00431-020-03659-8 (PMC7547982; doi:10.1007/s00431-020-03659-8)
Supplement: Supplementary file 2 — (PDF 123 kb) [file 431_2020_3659_MOESM2_ESM.pdf]

TABLE V. Initial (0h) vs. repeat laboratory studies (36-72h)

			Initial labs (0h)		Repeat labs 36-72h		
			Values	NA	Values	NA	p
Number of infections	ALL		105		105		
WBC (GPt/L)	median	(range)	9.47 (0-39)	0	13.96 (0-57)	25	0.022
WBC <5 or >21 GPt/L	n	(%)	40 (38%)	0	26 (33%)	25	NS
ITQ >0.2	n	(%)	63 (67%)	11	22 (31%)	33	<0.001
CrP (mg/L)	median	(range)	11 (0-294)	2	49.9 (0-315)	32	<0.001
CrP >10mg/L	n	(%)	53 (51%)	2	57 (78%)	32	<0.001
All labs normal *	n	(%)	18 (18%)	3	9 (11%)	25	NS
Number of infections	GBS		33		33		
WBC (GPt/L)	median	(range)	12.2 (2-33)	0	14.11 (2-57)	6	NS
WBC <5 or >21 GPt/L	n	(%)	14 (42%)	0	9 (33%)	6	NS
ITQ >0.2	n	(%)	23 (74%)	2	4 (17%)	9	<0.001
CrP (mg/L)	median	(range)	9.9 (0-150)	0	37.9 (2-315)	8	0.024
CrP >10mg/L	n	(%)	16 (48%)	0	19 (76%)	8	NS
All labs normal *	n	(%)	7 (21%)	0	2 (7%)	5	NS
Number of infections	E. coli		73		73		
WBC (GPt/L)	median	(range)	9.12 (0-39)	0	13.96 (0-52)	19	NS
WBC <5 or >21 GPt/L	n	(%)	26 (36%)	0	18 (33%)	19	NS
ITQ >0.2	n	(%)	41 (64%)	9	18 (37%)	24	0.005
CrP (mg/L)	median	(range)	12.25 (0-294)	2	49.9 (0-292)	24	0.003
CrP >10mg/L	n	(%)	38 (54%)	2	38 (78%)	24	0.008
All labs normal *	n	(%)	11 (15%)	1	7 (13%)	19	NS

WBC White blood cells
ITQ Immature/total quotient
CrP C-reactive protein
IL-6 Interleukin-6
* WBC 5-21, IT<0.2, CrP <10

Statistical analyses was formed with Kruskal-Wallis test for continuous covariates and Fisher's exact tests for categorical variables. P-values of ≤0.05 were deemed to be significant.
